# Supplementary material for: Complex‐centric proteome profiling by SEC‐SWATH‐MS
Source: Mol Syst Biol. 2019 Jan 14;15(1):e8438. doi: 10.15252/msb.20188438 (PMC6346213; doi:10.15252/msb.20188438)
Supplement: Supplementary file 8 — Dataset EV7 [file MSB-15-e8438-s008.zip › feature_plots_string/P82932.pdf]

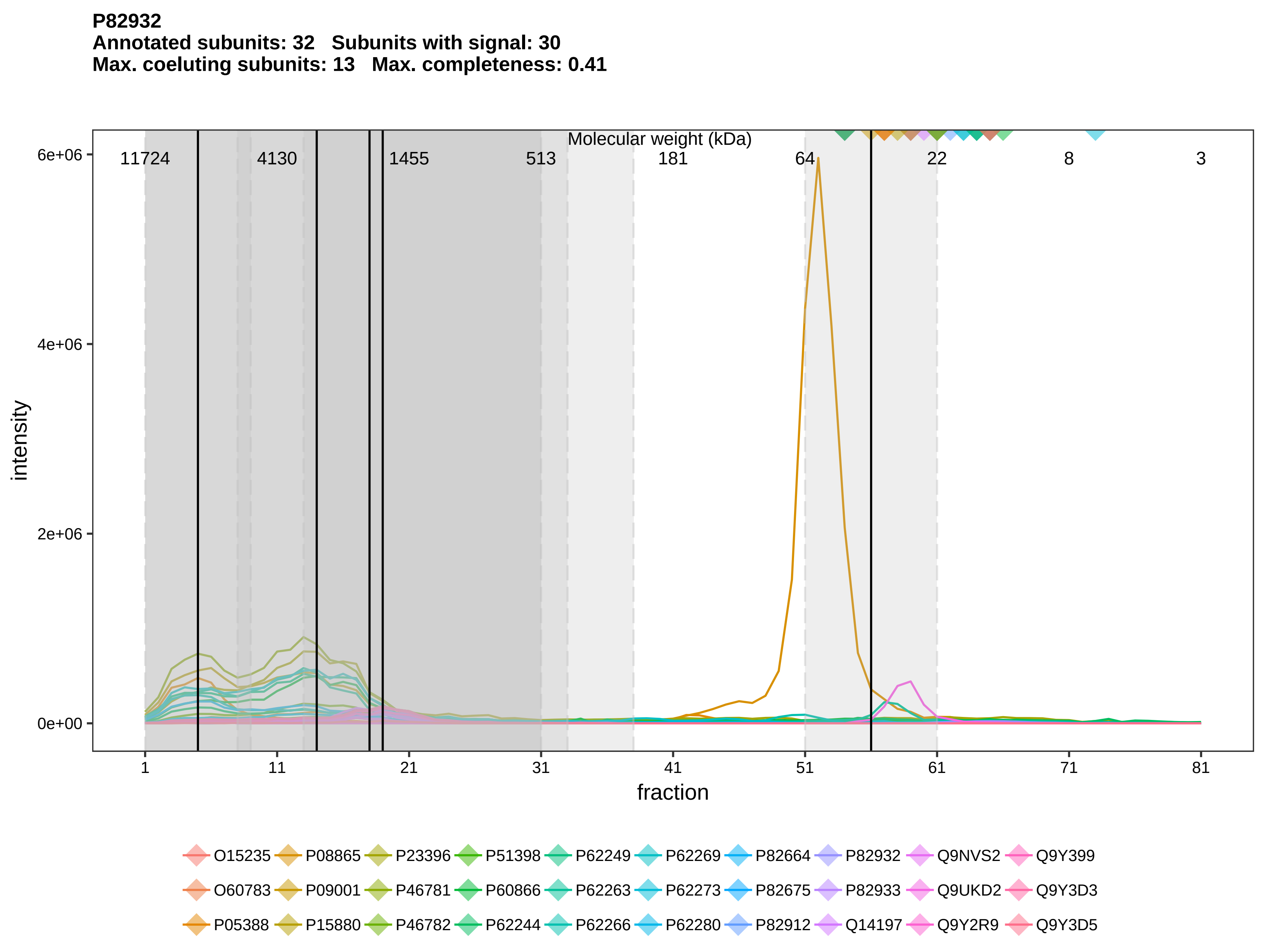
P82932
Annotated subunits: 32   Subunits with signal: 30
Max. coeluting subunits: 13   Max. completeness: 0.41
Molecular weight (kDa)
11724
4130
1455
513
181
64
22
8
3
intensity
6e+06
4e+06
2e+06
0e+00
fraction
1
11
21
31
41
51
61
71
81
O15235
P08865
P23396
P51398
P62249
P62269
P82664
P82932
Q9NVS2
Q9Y399
O60783
P09001
P46781
P60866
P62263
P62273
P82675
P82933
Q9UKD2
Q9Y3D3
P05388
P15880
P46782
P62244
P62266
P62280
P82912
Q14197
Q9Y2R9
Q9Y3D5
